# Split anergized natural killer cells halt inflammation by inducing stem cell differentiation, resistance to NK cell cytotoxicity and prevention of cytokine and chemokine secretion

**DOI:** 10.18632/oncotarget.3250

**Published:** 2015-03-27

**Authors:** Han-Ching Tseng, Nicholas Cacalano, Anahid Jewett

**Affiliations:** ^1^ Division of Oral Biology and Oral Medicine, The Jane and Jerry Weintraub Center for Reconstructive Biotechnology, Los Angeles, CA, USA; ^2^ Department of Radiation Oncology, Division of Molecular and Cellular Oncology UCLA School of Medicine, Los Angeles, CA, USA; ^3^ The Jonsson Comprehensive Cancer Center, UCLA School of Dentistry and Medicine, Los Angeles, CA, USA

**Keywords:** IFN-γ, NK, OSCSCs, cytotoxicity, regulatory NK

## Abstract

The mechanism of suppression of NK cytotoxicity in cancer patients is not clearly established. In this paper we provide evidence that anergized NK cells induce differentiation of healthy Dental Pulp Stem Cells (DPSCs) or transformed Oral Squamous Cancer Stem Cells (OSCSCs) resulting in cell growth inhibition, resistance to NK cell-mediated cytotoxicity and prevention of inflammatory mediators secretion. Induction of cytotoxicity resistance in differentiated cells correlated with increased CD54 and MHC class I surface expression and mediated by the combination of IFN-γ and TNF-α since antibodies to both, but not each cytokine alone, was able to inhibit resistance. In contrast, inhibition of cytokine and chemokine release was mediated by IFN-γ since the addition of anti-IFN-γ antibody, and not anti-TNF-α, restored secretion of inflammatory mediators in NK cell cultures with differentiated DPSCs and OSCSCs. There was a gradual and time dependent decrease in MHC class I and CD54 expression which correlated with the restoration of NK cell cytotoxicity, augmentation of cytokine secretion and increased cell growth from days 0–12 post NK removal. Continuous presence of NK cells is required for the maintenance of cell differentiation since the removal of NK cell-mediated function reverses the phenotype and function of differentiated cells to their stem-like cells.

## INTRODUCTION

Immune effectors such as Natural Killer cells and T cells are thought to shape tumor cells and select for cancers with reduced immunogenicity and enhanced capacity to induce immunosuppression [1]. Similarly, they are responsible for the selection of healthy stem cells with enhanced capacity to induce immunosuppression in order to mediate wound healing, tissue regeneration and cessation of inflammation. Although such mechanisms are thought or speculated to be the underlying cause of immunosuppression in tumors no direct evidence, clear data or even physiological relevance has previously been offered to demonstrate the rationale for tumor mediated immunosuppression. In this paper we provide evidence for the potential role of NK cells in selection, differentiation and cessation of inflammation either during their interaction with healthy stem cells (Supplementary data) or when interacting with tumors. This report provides the basis for the understanding of why immunosuppression in tumors may be a key mechanism in controlling tumor growth, even though such mechanism can be a double edge sword. In one hand it will control the growth and expansion of the tumors and in other hand it will allow the survival of a selected subpopulation of tumors. Any intervention or strategy to eliminate tumors should consider both mechanisms since they are interrelated.

Much work has been done to identify strategies by which tumor cells evade the immune system [2–7]. It has been shown that freshly isolated tumor infiltrating NK cells are not cytotoxic to autologous tumors. Furthermore, NK cells obtained from the peripheral blood of patients with cancer have significantly reduced cytotoxic activity [8–11]. In addition, NK cell cytotoxicity is suppressed after their interaction with stem cells [12–14]. In contrast, interaction of NK cells with the resistant tumors does not lead to suppression of NK cell cytotoxicity [15]. Moreover, two key transcription factors, NFκB and STAT3, were identified and shown to increase the production of multiple tumor-derived immunosuppressive molecules [16]. Undoubtedly, the same mechanisms are likely important for normal tissue regeneration and induction of resistance to NK and T cell mediated cytotoxicity.

We have previously shown that K562, an NK sensitive tumor, causes loss of NK cell cytotoxicity while increasing IFN-γ secretion by the NK cells [15, 17]. On the other hand NK resistant tumors such as RAJI cells do not induce loss of NK cell cytotoxicity nor trigger IFN-γ secretion [15, 17]. Significant down-modulation of CD16 receptor expression and decreased NK cell cytotoxic function were seen in oral and ovarian cancer patients [18, 19]. In addition, down-regulation of CD16 surface receptors on NK cells was also observed when NK cells were cultured with ovarian tumors [20]. The decrease in CD16 surface receptors was accompanied by a major decrease in NK cell killing activity against K562 tumor cells [20]. Triggering of CD16 on NK cells by anti-CD16 antibody, which mimics the ligand binding effect, was also found to result in down-modulation of CD16 receptors, a great loss of cytotoxicity, and gain in cytokine secretion in NK cells which we have previously coined as “split anergy” [15, 17, 21–25]. In addition, a small subpopulation of NK cells undergoes cell death similar to that seen during the interaction of NK cells with sensitive tumors [23, 25].

We previously demonstrated that NK resistant differentiated primary oral epithelial tumors, unlike their cancer initiating or stem cells, demonstrate higher nuclear NFκB activity and secrete significant levels of GM-CSF, IL-1β, IL-6 and IL-8 [26, 27]. Additionally, inhibition of NFκB in tumors leads to a significant increase in NK cell mediated cytotoxicity and augmented secretion of IFN-γ [28, 29]. Moreover, targeted inhibition of NFκB in distinct cells results in the induction of auto-immunity and inflammation [30, 31].

We have previously shown that stem-like oral tumors are significantly more susceptible to NK cell mediated cytotoxicity; whereas, their differentiated counterpart OSCCs is significantly more resistant [27]. In addition, hESCs, hiPSCs, hMSCs and hDPSCs, were found to be significantly more susceptible to NK cell mediated cytotoxicity than their differentiated counterparts [27]. Therefore, we proposed and recently demonstrated that NK cells play a significant role in differentiation of the cells by providing critical signals via secreted cytokines as well as direct cell-cell contact [32]. In addition, we have shown previously that monocytes, a subset of Myeloid Derived Suppressor Cells (MDSCs) induce significant split anergy in NK cells [33–37]. Such alterations in NK cell effector function is found to ultimately aid in driving differentiation of surviving, healthy, as well as transformed stem cells [33–37]. In cancer patients with advanced disease since the number and function (both the cytotoxic and cytokine secretion) of NK cells may be compromised by the growth and expansion of cancer stem cells, they may not be effective in eliminating and/or differentiating cancer stem cells, thus resulting in the progression of cancer. In this paper we demonstrate that anergized NK cells contribute to the differentiation and resistance to NK cell mediated cytotoxicity of transformed stem cells by secreting key cytokines. More importantly, we also demonstrate that NK differentiated stem cells not only resist lysis by the NK cells but also they do not trigger secretion of cytokines or chemokines, potentially contributing to the inhibition of inflammation. Such effects by the NK cells may allow the repair of the tissues during normal wound healing whereas during tumorigenesis they may aid in decreasing growth, invasion and metastasis of tumors, while allowing survival of a selected tumor population.

## RESULTS

### Resistance of differentiated but not stem-like tumors to NK cell mediated cytotoxicity

To determine whether NK cells target cancer stem cells and not their differentiated counterparts, NK cells were left untreated or treated with anti-CD16 antibody and/or IL-2 for 18–24 hours to induce split anergy before they were used in cytotoxicity assays against OSCSCs and OSCCs. As shown previously and in the Supplementary Figure 1, NK cells mediated much higher lysis of stem like OSCSCs when compared to differentiated OSCCs (*P* < 0.05) (Supplementary Figure 1A) [27]. OSCSCs were found to express a number of stem cell markers and they were CD133^+^CD44^+^CD326^+^CD26^+^CD338^+^CD166^dim^ [27, 38–41]. Both untreated and IL-2 treated NK cells mediated higher lysis of OSCSCs when compared to OSCCs in ^51^Cr release assay (*P* < 0.05) (Supplementary Figure 1A) [27] and IL-2 treated NK cells secreted higher levels of IFN-γ in co-culture with OSCSCs when compared to OSCCs (*P* < 0.05) (Supplementary Figure 1B) [27]. Anti-CD16mAb treatment inhibited NK cell cytotoxicity against both OSCSCs and OSCCs; however it did not induce much secretion of IFN-γ (Supplementary Figure 1) [27]. The addition of the combination of IL-2+anti-CD16mAb treatment, although significantly inhibited NK cell cytotoxicity against OSCSCs and OSCCs when compared to IL-2 activated NK cells (*P* < 0.05) (Supplementary Figure 1A), it induced much higher release of IFN-γ when cultured in the presence and absence of OSCSCs (Supplementary Figure 1). The levels of IFN-γ secretion remained less in the co-cultures of IL-2 or IL-2+anti-CD16mAb treated NK cells with OSCCs when compared to those cultured with OSCSCs (*P* < 0.05) (Supplementary Figure 1). Therefore, anti-CD16mAb in combination with IL-2 induced split anergy in NK cells resulting in a loss of cytotoxicity but gain in secretion of IFN-γ against oral stem-like tumors (Supplementary Figure 1). Similar results to those obtained with OSCSCs and OSCCs were also obtained with healthy untransformed primary Dental Pulp Stem Cells (DPSCs) and their differentiated counterpart (data not shown) and [27]. Noteworthy, IL-2 treated NK cells mediated much higher lysis of undifferentiated DPSCs when compared to differentiated DPSCs and the addition of the combination of IL-2+anti-CD16mAb treatment, although inhibited NK cell cytotoxicity against undifferentiated and differentiated DPSCs, it induced higher release of IFN-γ [27].

### Supernatants from the combination of IL-2+anti-CD16mAb treated NK cells induced resistance of OSCSCs to NK cell mediated cytotoxicity

**Figure 1:**
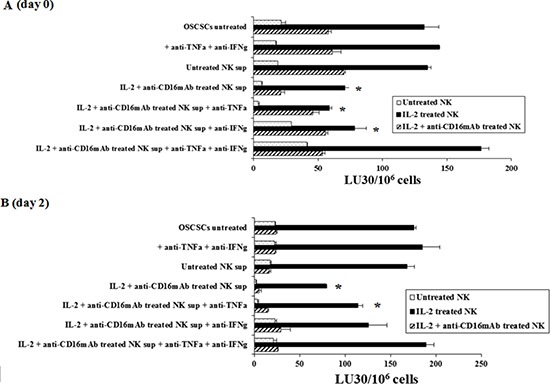
Induction of resistance to NK cell mediated lysis of OSCSCs treated with IL-2+anti-CD16mAb NK cells supernatant is mediated by the combination of IFN-γ and TNF-α and not each cytokine alone Highly purified NK cells were left untreated or treated with the combination of IL-2 (1000 units/ml) and anti-CD16mAb (3 μ g/ml) for 24 hours, after which the supernatants were removed and used for the treatment of OSCSCs. Untreated OSCSCs and those treated with anti-TNF-α (1:100) and anti-IFN-γ (1:100) in the absence of NK supernatants were also used as controls. Same amounts of supernatants from untreated NK cells and those cultured with IL-2+anti-CD16mAb treated NK cells in the presence and absence of anti-TNF-α (1:100) and/or anti-IFN-γ (1:100) were used to treat OSCSCs for a period of 4 days to induce differentiation. Differences between untreated OSCSCs and those stimulated with IL-2+ anti-CD16mAb treated NK supernatants with or without the addition of either anti-TNF-α or anti-IFN-γ alone were significant at a *p* value of < 0.05 (*). **(A)** OSCSCs were treated as described in Figure 1A for a period of 4 days before they were washed extensively and cultured in medium in the absence of NK supernatants for 2 days. Differences between untreated OSCSCs and those stimulated with IL-2+ anti-CD16mAb treated NK supernatants with or without the addition of anti-IFN-γ alone were significant at a *p* value of < 0.05 (*). **(B)** The cytotoxicity against untreated OSCSCs and those treated with anti-TNF-α and anti-IFN-γ in the absence of NK supernatants, and OSCSCs cultured with either untreated NK supernatants or those cultured with the supernatants from IL-2 + anti-CD16mAb treated NK cells in the presence and absence of antibodies to TNF-α and IFN-γ were assessed using untreated, IL-2 treated and the combination of IL-2+anti-CD16mAb treated freshly isolated NK cells using a standard 4 hour ^51^Cr release assay. Percent cytotoxicity was obtained at different effector to target ratio, and the lytic units 30/10^6^ cells were determined using inverse number of NK cells required to lyse 30% of the tumor cells X100.

To determine whether supernatants from split anergized NK cells are capable of inducing differentiation in OSCSCs, NK cells were left untreated or treated with anti-CD16 antibody and IL-2 for 18–24 hours before their supernatants were removed and added to OSCSCs. In addition, we determined the period of time which was required for the NK differentiated tumors to regain sensitivity to NK cell mediated cytotoxicity after the removal of NK supernatants. Treatment of OSCSCs with IL-2+anti-CD16mAb treated NK cell supernatants, but not untreated NK supernatants, for 4 days decreased NK cell mediated cytotoxicity significantly by freshly isolated untreated or IL-2 treated NK cells (*P* < 0.05) (Figure 1A). Resistance of OSCSCs to NK cell mediated cytotoxicity could also be observed after their treatment with supernatants from IL-2 treated NK cells, however, the levels of resistance were significantly less when compared to those induced by IL-2+anti-CD16mAb treated NK cell supernatants correlating with the degree of differentiation based on the surface receptor expression [32].

To examine the mechanisms by which OSCSCs become resistant by anergized NK cells, we determined NK cell mediated cytotoxicity when OSCSCs were treated with supernatants of NK cells treated with anti-CD16mAb and IL-2 in the presence and absence of each of IFN-γ and TNF-α antibodies alone or their combination. As shown in Figure 1A the addition of each of the TNF-α and IFN-γ antibody alone had no or a very slight inhibitory effect on the induction of resistance of OSCSCs on day zero or day 2 after the removal of the NK supernatants respectively, however, the combination of anti-IFN-γ and anti-TNF-α abrogated the resistance of treated OSCSCs completely (Figure 1A). The inhibition of OSCSCs resistance to NK cell mediated cytotoxicity by the combination of anti-IFN-γ and anti-TNF-α antibodies could be observed when untreated, IL-2 treated or IL-2+anti-CD16mAb treated NK cells (Figure 1A) were used to assess cytotoxicity. Treatment of OSCSCs with the combination of anti-TNF-α and anti-IFN-γ in the absence of NK supernatants had no effect on NK cell cytotoxicity (Figure 1A). Similar results to those shown above was also obtained when the supernatants of NK cells were removed from the OSCSCs and they were cultured in media for 2–6 days before they were used in cytotoxicity assay against NK cells. The levels of resistance of OSCSCs to NK cell mediated cytotoxicity were gradually decreased from day 0 to day 2 (Figure 1B) and to day 6 (data not shown). Day 0 demonstrated the highest resistance, followed by day 2 in which the levels of resistance still remained substantial and by day 6 only 10%–20% resistance could be observed. At day 12 post supernatant removal no differences were observed between IL-2+anti-CD16mAb supernatant treated OSCSCs and those cultured with supernatants from untreated NK cells (data not shown). Similar results to those seen with OSCSCs were also observed for DPSCs (Supplementary Figure 2A).

### Induction of resistance to NK lysis in OSCSCs by supernatants from IL-2+anti-CD16mAb treated NK cells correlated with the increased expression of CD54 and MHC class I

**Figure 2:**
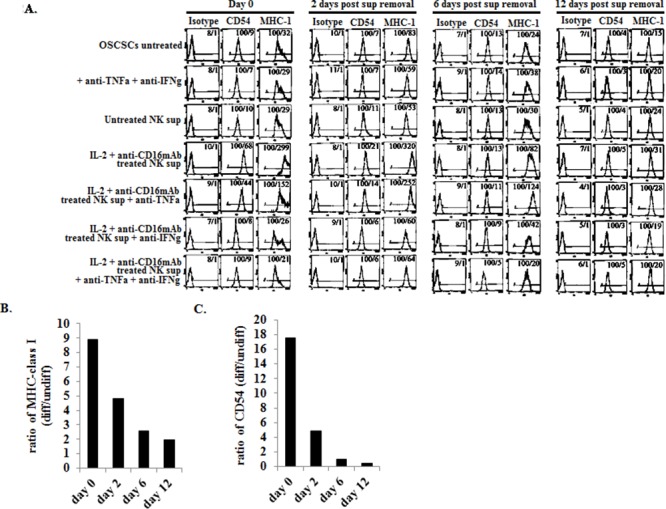
Increased expression of CD54 and MHC class I on OSCSCs differentiated with supernatants from IL-2+anti-CD16mAb treated NK cells OSCSCs were treated with NK supernatants in the presence and absence of anti-TNF-α and anti-IFN-γ antibodies as described in Figure 1A and the surface expression of CD54 and MHC Class 1 on untreated and NK supernatant treated OSCSCs were assessed after 4 days of differentiation. After differentiation, OSCSCs were washed and cultured in normal culture medium without the addition of NK supernatants for a period of 2 days, 6 days and 12 days. **(A)** Surface expression of CD54 and MHC Class I at each time point was assessed after PE conjugated antibody staining followed by flow cytometric analysis. Isotype control antibodies were used as controls. The numbers on the right hand corner are the percentages and the mean channel fluorescence intensities in each histogram. The ratios of MHC class I **(B)** or CD54 **(C)** at days 0, 2, 6 and 12 were determined by using the mean channel fluorescence of the IL-2+anti-CD16mAb treated NK supernatant differentiated OSCSCs to the mean channel fluorescence of the untreated OSCSCs.

We then compared NK cell resistance induced by the supernatants from IL-2+anti-CD16mAb treated NK cells in OSCSCs to expression of key cell surface receptors before and after removal of NK supernatants. Among many surface receptors tested, B7H1, CD44, CD54 and MHC class I expression were found to correlate significantly with the differentiation and resistance of OSCSCs to NK cell mediated cytotoxicity [32]. However, in this report we focused on only CD54 and MHC class I. As shown in Figure 2A the levels of CD54 and MHC class I increased substantially on OSCSCs in the presence of IL-2+anti-CD16mAb treated NK cell supernatants. Supernatants from untreated NK cells did not have significant effect on surface expression of OSCSCs (Figure 2A). The addition of a combination of anti-TNF-α and anti-IFN-γ antibodies at the initiation of OSCSCs treatment with IL-2+anti-CD16mAb treated NK supernatants prevented the up-regulation of CD54 and MHC class I on OSCSCs (Figure 2A). The effect of anti-IFN-γ antibody in the absence of anti-TNF-α antibody, was more dominant for surface receptor modulation than cytotoxicity or cell growth (please see below) since its addition abrogated the increase in CD54 and MHC class I on OSCSCs (Figure 2A). Similar results to those shown above were also obtained when the NK supernatants were removed from the OSCSCs on day 0 post-differentiation and they were replaced by media from day 0 to day 6 (Figures 2A–2C). The levels of MHC class I and CD54 surface receptors gradually decreased from day 0 to day 2 and then to day 6 post removal of NK cell supernatants (Figures 2A–2C). At day 12 post NK supernatant removal only 1.5–2 fold increase in MHC class I and 1–1.2 fold increase in CD54 expression could be observed on OSCSCs cultured with supernatants from IL-2+anti-CD16mAb treated NK cells (Figures 2A–2C). Thus, there was a time dependent decrease in the ratios for MHC class I and CD54 surface receptor expression between IL-2+anti-CD16mAb NK supernatant treated OSCSCs versus those cultured with supernatants from untreated NK cells or OSCSCs in the absence of any treatment (Figures 2B and 2C). Similar results to those seen with OSCSCs were also observed for DPSCs [32].

### Treatment of OSCSCs by supernatants from IL-2+anti-CD16mAb treated NK cells severely inhibited secretion of cytokines and chemokines by the NK cells

**Figure 3:**
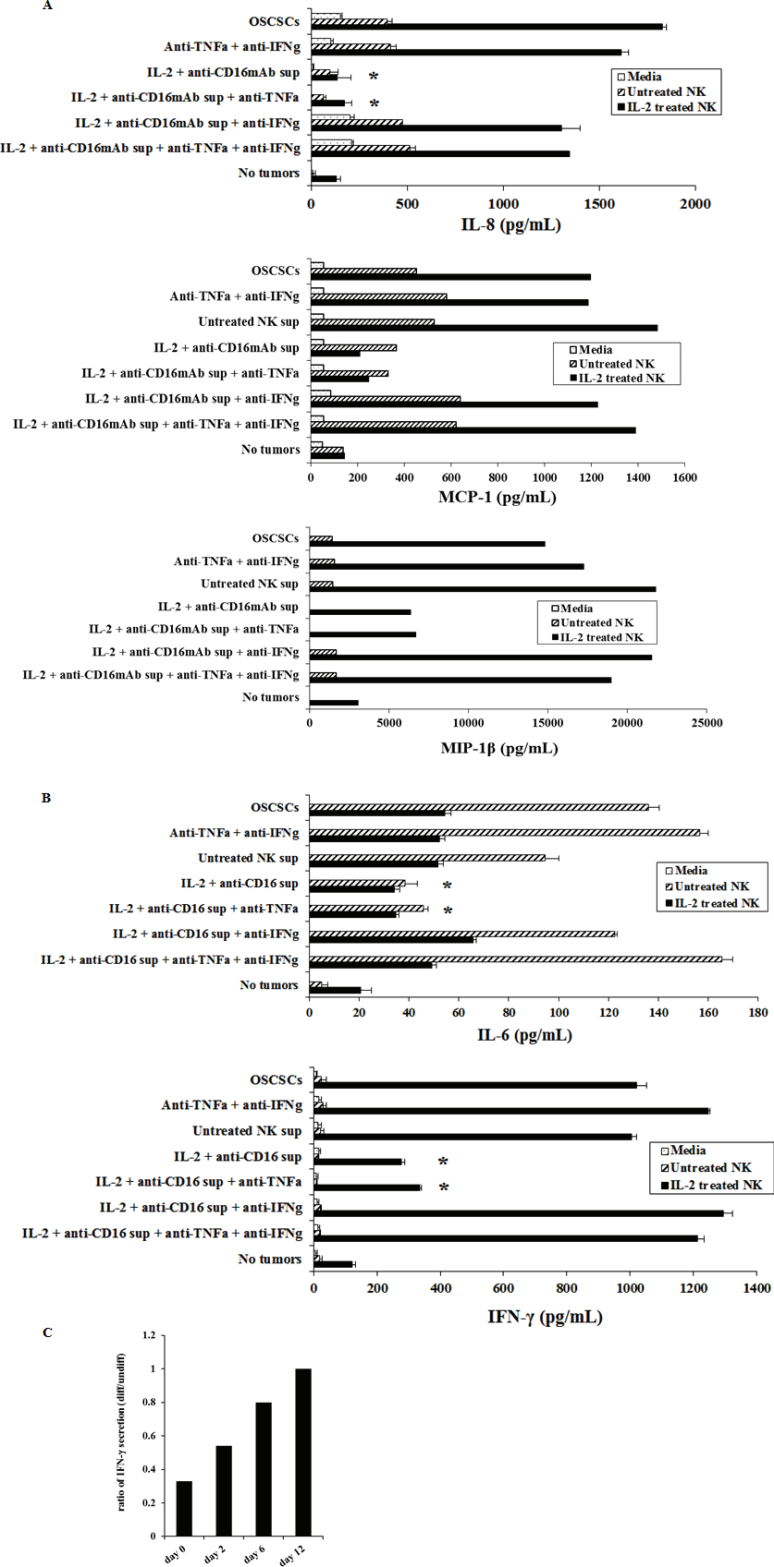
OSCSCs cultured with supernatants from IL-2+anti-CD16mAb treated NK cells significantly inhibited the production of IL-6 and IFN-γ cytokines and IL-8, MCP-1 and MIP-1β chemokines by NK cells Freshly isolated NK cells were left untreated or treated with IL-2 (1000 units/ml) for 18 hours. Afterwards, NK cells were added to OSCSCs treated with NK cell supernatants as described in Figure 1A at an effector to target ratio of 0.5 to 1. After an overnight incubation, the supernatants were removed from the co-cultures and the levels of IL-8, MCP-1, MIP-1β chemokines **(A)**, and IL-6 and IFN-γ cytokines **(B)**, secretions were determined using multiplexed Luminex ELISAs. Identical results for cytokines and chemokine secretion were obtained using either single or the multiplexed format. Differences between untreated OSCSCs and those stimulated with IL-2+anti-CD16mAb treated OSCSCs with or without the addition of anti-TNF-α were significant at a *p* value of < 0.05 (*). The ratios of IFN-γ production by IL-2 treated NK cells at day 0, 2, 6 and 12 post-differentiation were determined by comparing the amounts of IFN-γ secreted in the co-cultures of NK cells with differentiated OSCSCs with the supernatants from IL-2+anti-CD16mAb treated NK cells with those secreted in the co-cultures of NK cells with untreated OSCSCs **(C)**.

We next determined whether decrease in NK cell mediated cytotoxicity correlates with a decrease in cytokine and chemokine secretion in cultures of OSCSCs treated with supernatants from split anergized NK cells. In addition, we determined the period of time which was required for the NK differentiated tumors to regain sensitivity to NK cells and increase cytokine and chemokine secretion after the removal of NK supernatants. Treatment of OSCSCs with IL-2+anti-CD16mAb treated NK cell supernatants significantly decreased secretion of chemokines IL-8 (*P* < 0.05), MCP-1 and MIP1β (Figure 3A) and cytokines IL-6 (*P* < 0.05) and IFN-γ (*P* < 0.05) (Figure 3B) when cultured with freshly isolated untreated or IL-2 treated NK cells. To examine the mechanisms by which NK supernatant treated OSCSCs decrease cytokine secretion by NK cells, we determined secretion in the presence and absence of each of IFN-γ and TNF-α antibodies alone or their combination. As shown in Figure 3, the addition of TNF-α antibody in the absence of anti-IFN-γ to OSCSCs treated with IL-2+anti-CD16mAb NK supernatants had no or low effect on the increase in secretion of cytokines and chemokine. In contrast, the addition of anti-IFN-γ, in the absence of anti-TNF-α, increased the levels of IL-8, MCP-1, MIP-1β, IL-6 and IFN-γ in the co-cultures of NK cells with IL-2+ anti-CD16mAb treated OSCSCs to the levels when OSCSCs treated with the unstimulated NK supernatants were cultured with NK cells (Figure 3). The increase in the secretion of cytokines and chemokines in the co-cultures of NK cells with IL-2+anti-CD16mAb treated OSCSCs were also observed when they were cultured in the presence of both anti-TNF-α and anti-IFN-γ antibodies (Figure 3). Similar results to those shown above were also obtained when the supernatants of IL-2+anti-CD16mAb treated NK cells were removed from the OSCSCs and were replaced by media from day 0–6 post-differentiation (data not shown, and Figure 3C), however, the levels of cytokines gradually rose from day 0–6 and by day 12 post-differentiation could be seen between OSCSCs treated with the supernatants from IL-2+anti-CD16mAb stimulated NK cells and those cultured with untreated OSCSCs (Figure 3C). As the levels of CD54 and MHC class I gradually decreased from day 0–12 (Figure 2), the levels of IFN-γ secretion in the co-cultures of NK cells with IL-2+anti-CD16mAb NK supernatant differentiated OSCSCs gradually rose to the same levels obtained in the co-cultures of NK cells with OSCSCs cultured with supernatants from untreated NK cells (Figure 3C). Thus, there was a time dependent decrease in the expression of CD54 and MHC class I which correlated with restoration of cytokine secretion in co-cultures of NK cells with IL-2+anti-CD16mAb NK supernatant differentiated OSCSCs when NK supernatants were removed and replaced with media from day 0–12 (Figures 2, 3). Similar results to those seen with OSCSCs were also observed for DPSCs (Supplementary Figure 2B).

### Treatment of OSCSCs by supernatants from IL-2+anti-CD16mAb treated NK cells limited OSCSCs cell numbers

**Figure 4:**
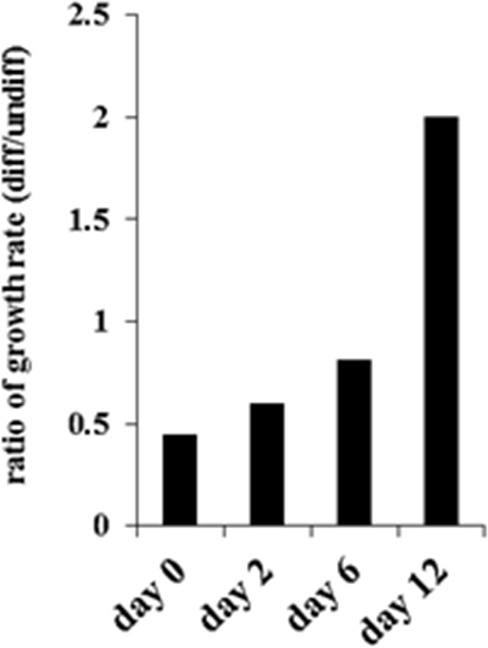
Decreased numbers of OSCSCs after treatment with supernatants from IL-2+anti-CD16mAb treated NK cells OSCSCs were treated with supernatants from IL-2+anti-CD16mAb treated NK cells for 4 days (day 0 of post-differentiation) after which treated OSCSCs were washed and cultured in media for an additional 2, 6 and 12 days. The ratios of tumor growth were determined by comparing the number of IL-2+anti-CD16mAb treated NK supernatant differentiated OSCSCs with untreated OSCSCs at each time point.

To determine growth dynamics of OSCSCs after treatment with the NK supernatants, the numbers of OSCSCs were counted after treatment with the NK cell supernatants by microscopic evaluation and the levels of cell death were determined by staining with propidium iodide followed by flow cytometric analysis. As shown in Figure 4 there was a decrease in the numbers of OSCSCs after their treatment with IL-2+anti-CD16mAb treated NK cell supernatants when compared to untreated OSCSCs or those cultured with untreated NK cell supernatants (Figure 4). In addition, the decrease in the rate of cell growth was completely inhibited in the presence of the combination of anti-IFN-γ and anti-TNF-α antibodies and not each antibody alone [32]. Interestingly, at day 12 of post supernatant removal the rate of growth in OSCSCs treated with supernatants from IL-2+anti-CD16mAb treated NK cells increased 2 fold when compared to OSCSCs cultured either with untreated NK cell supernatants or untreated OSCSCs (Figure 4). Moreover, when the viability of OSCSCs were determined after the addition of supernatants from the IL-2+anti-CD16mAb treated NK cells, no significant cell death in the OSCSCs were seen at day 0 (Supplementary Figure 3) or at days 2–12 after supernatant removal (data not shown).

## DISCUSSION

In this paper we provide evidence that anergized NK cells have the ability to induce differentiation of cancer stem cells and limit inflammation through the release of TNF-α and IFN-γ. Similar results to those observed with supernatants were also obtained when fixed IL-2+anti-CD16mAb treated NK cells were used to differentiate OSCSCs [32]. In addition, monensin treated and fixed NK cells lost the ability to induce resistance and differentiation of OSCSCs [32].

Differentiation of OSCSCs by anergized NK cells inhibited greatly the secretion of cytokines and chemokines in the cultures of NK cells with differentiated tumors. This observation is of great significance since it may indicate that cellular differentiation is an important step in inhibition of inflammation. Indeed, the levels of cytokines and chemokines secreted in the co-cultures of NK cells with anergized NK supernatant differentiated OSCSCs was in general similar or slightly higher than those secreted by the NK cells in the absence of tumors. Another intriguing observation in our previous studies is inhibition of bFGF in stem cells by anergized NK cells [27]. Since bFGF is important for the maintenance of stemness in a variety of cell types, inhibition of bFGF by anergized NK cells may be one of the mechanisms by which NK cells prevent growth and proliferation of stem cells and promote their differentiation [42, 43].

We also determined whether differentiation of OSCSCs with supernatants from anergized NK cells were long or short lived. There was a gradual and time dependent decrease in the expression of both MHC class I and CD54 which correlated with the increased cell growth and restoration of NK cell cytotoxicity and cytokine secretion in cultures of NK cells with differentiated OSCSCs from days 0–12 post NK supernatant removal. These experiments indicated that in order for OSCSCs to remain differentiated, a continuous exposure to cytokines are necessary since after their removal the cells revert to their undifferentiated phenotype and become sensitive to NK cell mediated cytotoxicity, and trigger the release of cytokines and chemokines. It is also possible that a few undifferentiated OSCSCs were able to escape differentiation when exposed to the anergized NK cell supernatant, and thus they were able to expand after the removal of NK cell cytokines. However, this possibility is less likely since all of the cells at the peak of differentiation were MHC class I and CD54 positive indicating that all of the cells had gone through differentiation and upon removal of cytokines secreted by NK cells they reverted to an undifferentiated phenotype. These results may explain the mechanisms underlying chronic auto-immune inflammation in which the patients suffer from the bouts of exacerbation and remission. Such plasticity in differentiated tumors may explain the need for continuous presence of immune cells in the tumor microenvironment for the inhibition of tumor invasion and metastasis. Indeed, patients which have tumors with infiltrating immune cells have a better prognosis than those which lack infiltration of immune effectors.

Lack of NK cytotoxic function against differentiated tumors may be due to the release of immunosuppressive cytokines such as TGF-β and IL-10 which regulate cytotoxicity as well as TNF-α and IFN-γ release. Our recent studies indicated that IL-10 is an important regulator which limits NK cell mediated tumor differentiation through inhibition of IFN-γ secretion during monocyte mediated induction of NK anergy (manuscript in prep). The potential role of TGF-β on NK cell mediated differentiation should await future studies.

It is interesting to note that anergized NK differentiated OSCSCs express higher levels of CD54; however, they are not susceptible to NK cell mediated cytotoxicity even though the increase in CD54 expression on tumors is shown to increase NK cell mediated cytotoxicity. It is clear from these experiments that CD54 binding and function in cytotoxicity may be limited depending on the differentiation status of the tumors.

OSCSCs do not secrete IL-6, however, the levels of IL-6 secretion are significantly elevated in the cultures of untreated NK and less with IL-2 treated NK cells with OSCSCs. This increase could be due to the elevated IL-6 release by the untreated NK cells, by OSCSCs or both after NK-tumor interaction and cross-signaling. Untreated or IL-2 treated NK cells trigger less IL-6 secretion when cultured with NK differentiated OSCSCs.

**Figure 5:**
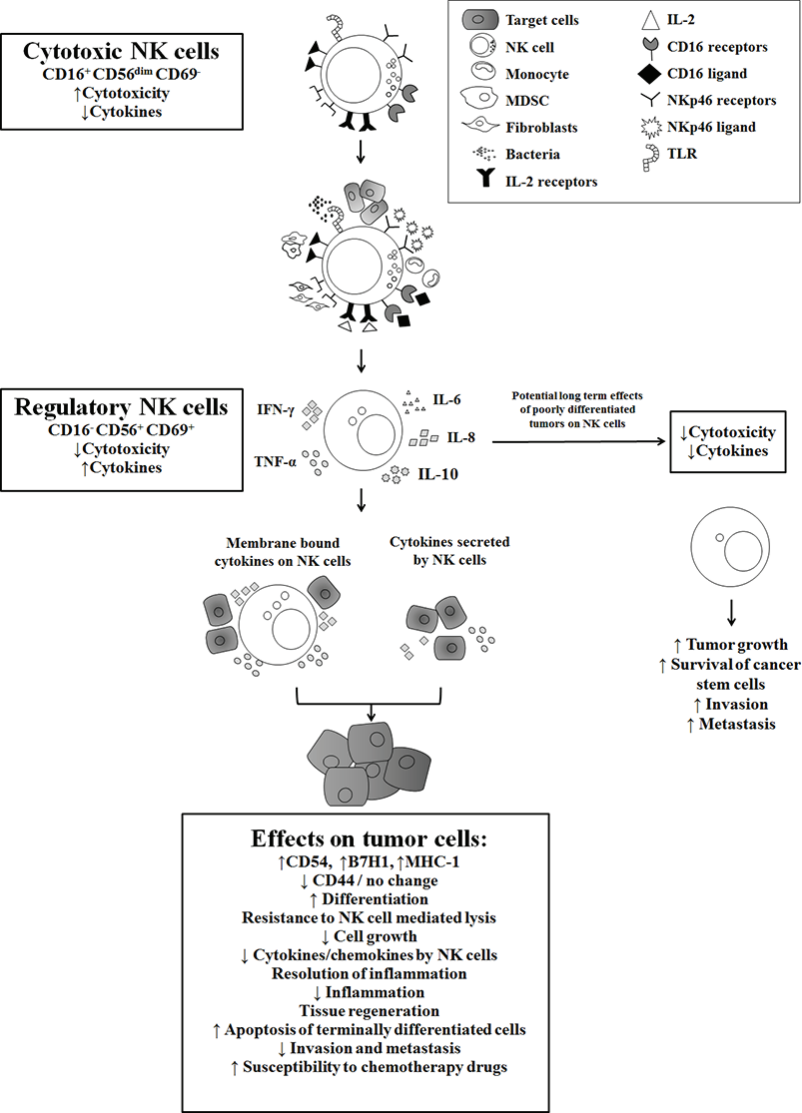
Hypothetical model of induction of anergized/regulatory NK cells in oral microenvironment by immune inflammatory cells and by the effectors of connective tissue to support differentiation of cancer stem cells resulting in their resistance to NK cell mediated cytotoxicity Induction of NK cell anergy in oral microenvironment is shown. Significant infiltration of immune effectors are likely to anergize NK cells to lose cytotoxicity and gain the ability to secrete cytokines, a term which we previously coined ‘split anergy’ in NK cells, and to support differentiation of stem cells. NK cells are likely to encounter and interact with other immune effectors such as monocytes/macrophages, other myeloid-derived suppressor cells (MDSCs) or with cancer-associated fibroblasts in oral microenvironment, in order to be conditioned to form anergized/regulatory NK (NKreg) cells. NK cells may also directly interact with stem cells at the base of the epithelial layer in oral-gingival tissues, in which case by eliminating their bound stem cells, they can become conditioned to support differentiation of other stem cells. NK cell-differentiated epithelial cells will no longer be killed or induce cytokine secretion by the NK cells, resulting in the resolution of inflammation.

Induction of split anergy in NK cells is an important NK cell conditioning step responsible for the differentiation of cells during pathological processes. In tumors, since the generation and maintenance of cancer stem cells is chronically high, the majority of NK cells including those of the circulating NK cells, may become conditioned to support differentiation of the cells and as such the phenotype of NK cells in tumor microenvironment as well as in the peripheral blood may resemble that of the anergic NK cells [33, 35–37, 44]. Therefore, our results suggest two very important functions for the NK cells. One potential function of NK cells is to limit the number of stem cells and second to support differentiation of the stem cells. In respect to the oral squamous cell carcinomas since the majority of immune effectors are found at the connective tissue area (Figure 5) [33, 35–37, 44], it is likely that NK cells may first encounter and interact with other immune inflammatory cells. By interacting with monocytes or dendritic cells or perhaps MDSCs, NK cells could then be in a position to support differentiation of tumor cells since they will be conditioned to lose cytotoxicity and release cytokines and growth factors [27, 34, 36, 45]. In agreement, all of the immune effectors isolated from diseased oral tissues have CD69^+^ phenotype [33, 35–37, 44]. In addition, lack of significant infiltration of NK cells in the tumor nest and the localization of NK cells in the immune rich compartment surrounding the tumor also provides the means for the induction of split anergy in NK cells in the tumor microenvironment [44].

Addition of anti-IFN-γ along with supernatants from anergized NK cells to OSCSCs increased cytokine secretion while significantly inhibiting the increase in MHC class I or CD54 surface receptor expression, while it required the presence of both anti-IFN-γ and anti-TNF-α to restore NK cell cytotoxicity. Thus, the effect of anti-TNF-α was less prominent when assessed for surface receptor expression, cytokine secretion or cell growth of OSCSCs when added along with supernatants from anergized NK cells to OSCSCs. However, addition of the combination of rTNF-α and rIFN-γ was found to increase synergistically the cell surface receptor expression while decreasing cell growth in OSCSCs [45]. The differences observed with anti-TNF-α versus rTNF-α on OSCSC differentiation could be due to the limitations of the assay conditions with anti-TNF-α antibody and not inability of TNF-α to influence differentiation. The exact role and extent of TNF-α and IFN-γ influence on OSCSC differentiation when anergized NK supernatants are added to OSCSCs should await future studies and is likely influenced by the expression and function of TNF-α and IFN-γ receptors on OSCSCs [45].

There should be two distinct mechanisms for the elimination of tumors, one which targets stem cells and the other which targets differentiated cells. Since cancer stem cells are resistant to chemotherapeutic drugs but sensitive to NK cell mediated killing while differentiated oral tumors are more resistant to NK cell mediated killing but more susceptible to chemotherapeutic drugs, combination therapy should be effective for the elimination of tumors [32]. In addition, since a great majority of cancer patients’ NK cells are anergized, they may not be effective in killing cancer stem cells. Therefore, these patients should benefit from repeated allogeneic NK cell infusions from healthy individuals for the elimination of cancer stem cells. Indeed, our data indicates that continuous exposure to IFN-γ and TNF-α secreted by the NK cells is required for the maintenance of the tumors in a differentiated state, since the removal of NK supernatants from the differentiated tumors reverts the tumors to stem-like state within two weeks of the removal of NK supernatants. Both non-anergized and anergized NK cells are important for the containment of the tumors since each will contribute to the decrease in growth and expansion of cancer stem cells. Depletion of NK anergizing effectors such as monocytes in the tumor microenvironment may provide greater cytotoxicity, but they may also halt or decrease the ability of NK cells to drive differentiation of the tumors effectively since monocytes induce synergistic release of IFN-γ and TNF-α by the NK cells. In addition, a strong tumor differentiating microenvironment will ensure that most newly arising cancer stem cells are induced to differentiate (Figure 5). The benefit of this approach is the ability of chemotherapeutic drugs to target the differentiated tumors in addition to the lack of differentiated tumors to grow and metastasize. Indeed, our recent *in vivo* data indicated that pancreatic cancer stem cells have the ability to grow fast and metastasize, whereas their differentiated tumors grew slower and remained localized for a long period of time without metastasizing (manuscript in prep).

The most devastating outcome of the cancer is its ability to deplete the numbers and cytotoxicity and cytokine secretion capabilities of remaining NK cells and other immune inflammatory cells (Figure 5). In such cases, not only cancer stem cells survive but they remain poorly differentiated. Maintaining adequate numbers of NK cells in the patients is the key for prevention of tumor metastasis and invasion.

## MATERIALS AND METHODS

### Cell lines, reagents, and antibodies

RPMI 1640 supplemented with 10% Fetal Bovine Serum (FBS) (Gemini Bio-Products, CA) was used for the cultures of human NK cells. OSCCs and stem-like OSCSCs were isolated from the tongue tumors of the patients at UCLA and cultured in RPMI 1640 supplemented with 10% FBS (Gemini Bio-Products, CA), 1.4% antibiotic antimycotic, 1% sodium pyruvate, 1.4% non-essential amino acids, 1% L-glutamine, 0.2% gentamicin (Gemini Bio-Products, CA) and 0.15% sodium bicarbonate (Fisher Scientific, PA). The primary tumor cells are tested and authenticated regularly in our laboratory. DPSCs from patients were isolated from the third molars after tooth extraction at UCLA and they were cultured in DMEM complete medium supplemented with 2% FBS and 1% penicillin and streptomycin (Gemini Bio-Products, CA) and used with autologous NK cells.

Recombinant IL-2 was obtained from NIH-BRB. Recombinant TNF-α and IFN-γ were obtained from Biolegend (San Diego, CA). Antibody to CD16 was purchased from Biolegend (San Diego, CA). Anti-MHC class I were prepared in our laboratory and 1:100 dilution was found to be the optimal concentration to use. PE conjugated anti-CD54 and anti-CD44, were obtained from Biolegend (San Diego, CA). Monoclonal antibodies to TNF-α were prepared in our laboratory from ascites of mice injected with TNF-α hybridomas, after which the antibodies were purified and specificity determined by both ELISA and functional assays against recombinant TNF-α. Polyclonal IFN-γ antibodies were prepared in rabbits, purified and specificity determined with ELISA and functional assays against rIFN-γ. 1:100 dilution of anti-TNF-α and anti-IFN-γ antibodies were found to be the optimal concentration to block rTNF-α and rIFN-γ function. The human NK purification kits were obtained from Stem Cell Technologies (Vancouver, Canada). Propidium iodide is purchased from Sigma Aldrich (Buffalo, NY).

### Purification of NK cells

PBMCs from healthy donors were isolated as described before [15]. Briefly, peripheral blood lymphocytes were obtained after Ficoll-hypaque centrifugation and purified NK cells were negatively selected by using an NK cell isolation kit (Stem Cell Technologies, Vancouver, Canada). The purity of NK cell population was found to be greater than 90% based on flow cytometric analysis of anti-CD16 antibody stained cells. The levels of contaminating CD3+ T cells remained low, at 2.4% ± 1%, similar to that obtained by the non-specific staining using isotype control antibody throughout the experimental procedures. Written informed consents approved by UCLA Institutional Review Board (IRB) were obtained from the blood donors and all the procedures were approved by the UCLA-IRB.

### ELISA and multiplex assays

Single ELISAs were performed as described previously [15]. Fluorokine MAP cytokine multiplex kits were purchased from R&D Systems (Minneapolis, MN) and the procedures were conducted as suggested by the manufacturer. To analyze and obtain the cytokine and chemokine concentration, a standard curve was generated by either two or three fold dilution of recombinant cytokines provided by the manufacturer. Analysis was performed using the Star Station software.

### Surface staining and cell death assays

Staining was performed by labeling the cells with PE conjugated antibodies or propidium iodide as described previously [15, 46].

### ^51^Cr release cytotoxicity assay

The ^51^Cr release assay was performed as described previously [29]. Briefly, different numbers of purified NK cells were incubated with ^51^Cr–labeled tumor target cells. After a 4 hour incubation period the supernatants were harvested from each sample and counted for released radioactivity using the gamma counter. The percentage specific cytotoxicity was calculated as follows;

$$\%\text{ Cytotoxicity }=\;\frac{\text{Experimental cpm }-\text{ spontaneous cpm}}{\text{Total cpm }-\text{ spontaneous cpm}}$$

LU 30/10^6^ is calculated by using the inverse of the number of effector cells needed to lyse 30% of tumor target cells × 100.

### Stem cell differentiation with NK cell supernatant

Human NK cells were purified from healthy donor's PBMCs as described above. NK cells were left untreated or treated with anti-CD16mAb (3 ug/ml), IL-2 (1000 units/ml) or a combination or IL-2 (1000 units/ml) and anti-CD16mAb (3 ug/ml) for 18–24 hours before the supernatants were removed and used in differentiation experiments. The amounts of IFN-γ produced by the activated NK cells were assessed with IFN-γ ELISA (Biolegend, CA). Differentiation of OSCSCs was conducted with gradual daily addition of increasing amounts of NK cell supernatant. On average a total of 1000 pg of IFN-γ containing supernatants obtained from IL-2+anti-CD16mAb treated NK cells was added for 4 days to induce differentiation and resistance of OSCSCs to NK cell mediated cytotoxicity. DPSCs required on average a total of 3600 pg of IFN-γ containing supernatants obtained from IL-2+anti-CD16mAb treated NK cells during a 4 day treatment to promote differentiation and resistance to NK cell mediated cytotoxicity. Afterwards, target cells were rinsed with 1X PBS, detached and used for experiments.

### Statistical analysis

An unpaired, two-tailed student *t*-test was performed for the statistical analysis. One way ANOVA with a Bonferroni post-test was used to compare the different groups.

## SUPPLEMENTARY FIGURES


