# Supplementary material for: Split anergized natural killer cells halt inflammation by inducing stem cell differentiation, resistance to NK cell cytotoxicity and prevention of cytokine and chemokine secretion
Source: Oncotarget. 2015 Mar 27;6(11):8947–59. doi: 10.18632/oncotarget.3250 (PMC4496194; doi:10.18632/oncotarget.3250)
Supplement: Supplementary file 1 [file oncotarget-06-8947-s001.pdf]

SUPPLEMENTARY FIGURES

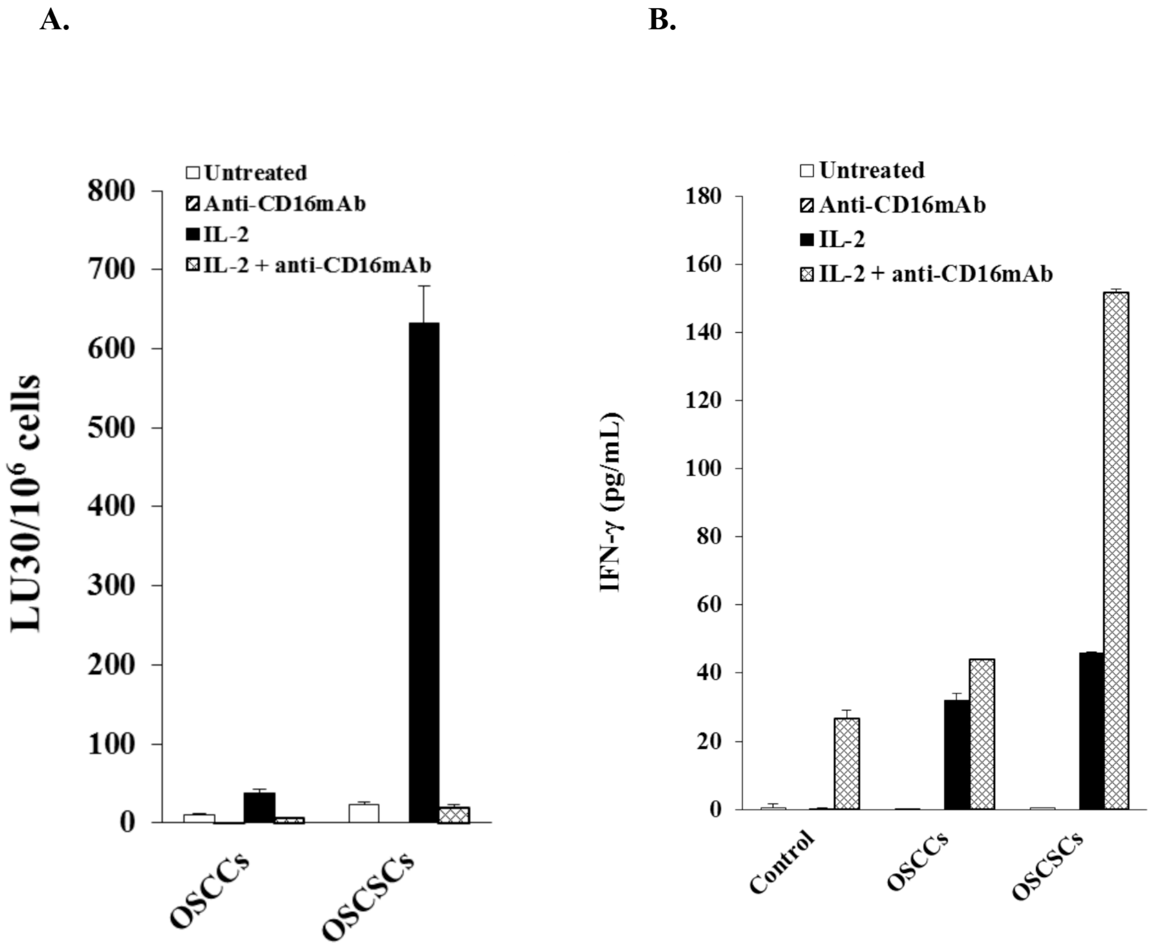

Supplementary Figure 1: NK cells are activated by OSCSCs but not by their differentiated OSCCs counterpart. NK cells were left untreated or treated with IL-2 (1000 units/ml), anti-CD16mAb (3 µg/ml) or a combination of IL-2 (1000 units/ml) and anti-CD16mAb (3 µg/ml) for 18 hours before they were added to ^{51}Cr labeled OSCSCs and OSCCs. NK cell mediated cytotoxicity was determined using a standard 4 hour ^{51}Cr release assay and the lytic units 30/10^{6} cells were determined using inverse number of NK cells required to lyse 30% of the target cells X100 **(A)**. NK cells were treated as described in Supplementary Figure 1A and each NK sample was cultured in the absence or presence of OSCSCs and OSCCs at an NK cell to target cell ratio of 0.5:1. After an overnight incubation, the supernatants were removed from the co-cultures and the levels of IFN-γ secretion were determined using specific ELISAs **(B)**. One of three representative experiments is shown in each figure.

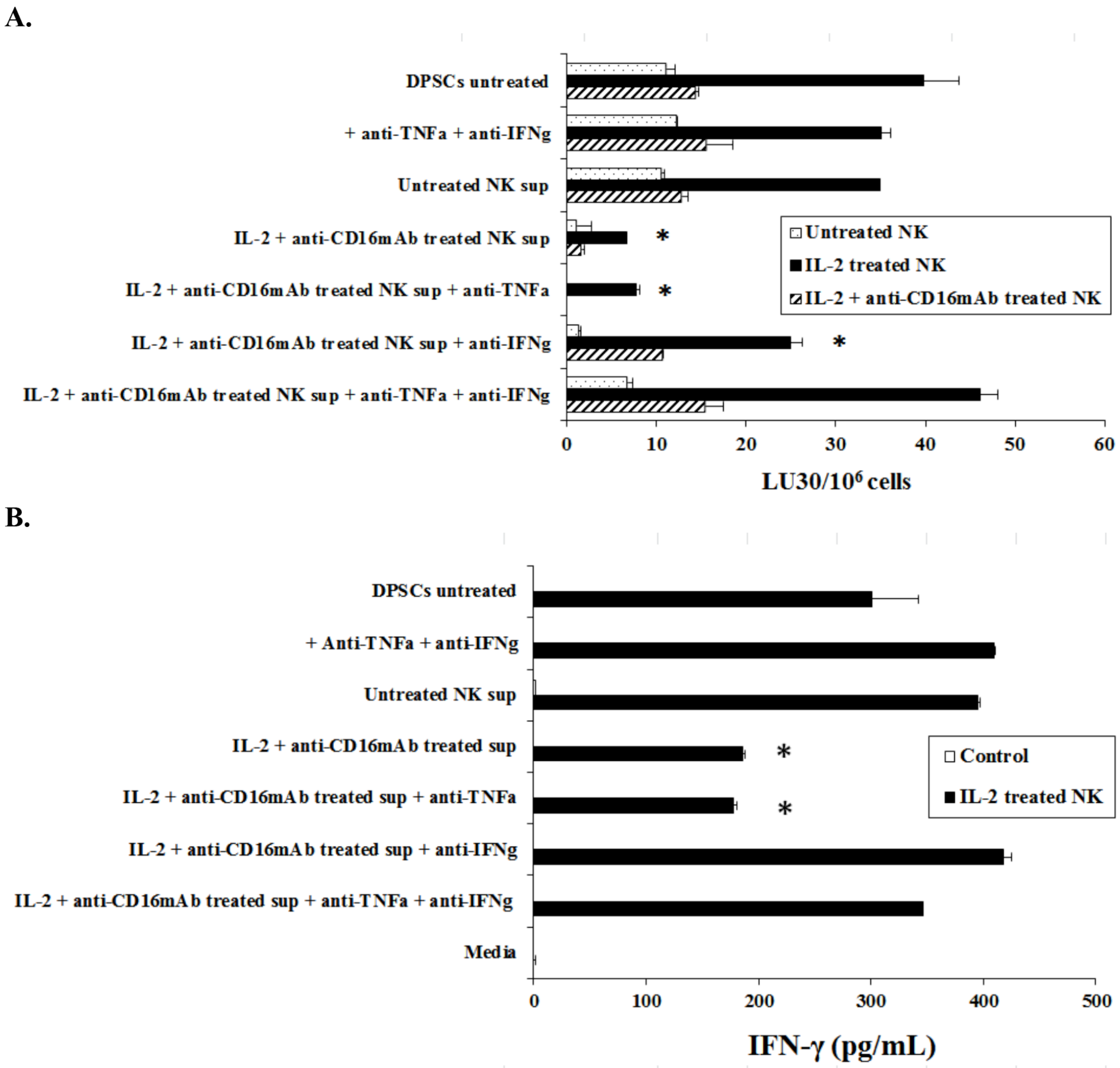

Supplementary Figure 2: Similar to OSCSCs, NK cells trigger autologous DPSCs differentiation resulting in their resistance to NK cell cytotoxicity and prevention of cytokine and chemokine secretion. Highly purified NK cells were left untreated or treated with the combination of IL-2 (1000 units/ml) and anti-CD16 mAb (3 µg/ml) for 18 hours, after which supernatants were removed and added to autologous DPSCs in the presence and absence of anti-TNF-α (1:100) and/or anti-IFN-γ (1:100) for a period of 4 days. The cytotoxicity of untreated, IL-2 treated and IL-2 + anti-CD16mAb treated NK cells against autologous NK supernatant treated DPSCs were assessed using a standard 4 hour ^{51}Cr release assay. Percent cytotoxicity was obtained at different effector to target ratio and the lytic units 30/10^6 cells were determined using inverse number of NK cells required to lyse 30% of the tumor cells X100. Differences between untreated DPSCs and those stimulated with IL-2+ anti-CD16mAb treated NK supernatants with or without the addition of either anti-TNF-α or anti-IFN-γ alone were significant at a p value of < 0.05 (*) **(A)**. Freshly isolated NK cells were left untreated or treated with IL-2 (1000 units/ml) for 18 hours. Afterwards, NK cells were added to DPSCs treated as described in Supplementary Figure 2A at an effector to target ratio of 0.5 to 1. After an overnight incubation, the supernatants were removed from the co-cultures and the levels of IFN-γ secretions were determined using specific ELISAs. Differences between untreated DPSCs and DPSCs stimulated with IL-2+ anti-CD16mAb treated NK supernatants with or without the addition of anti-TNF-α were significant at a p value of < 0.05 (*)**(B)**.

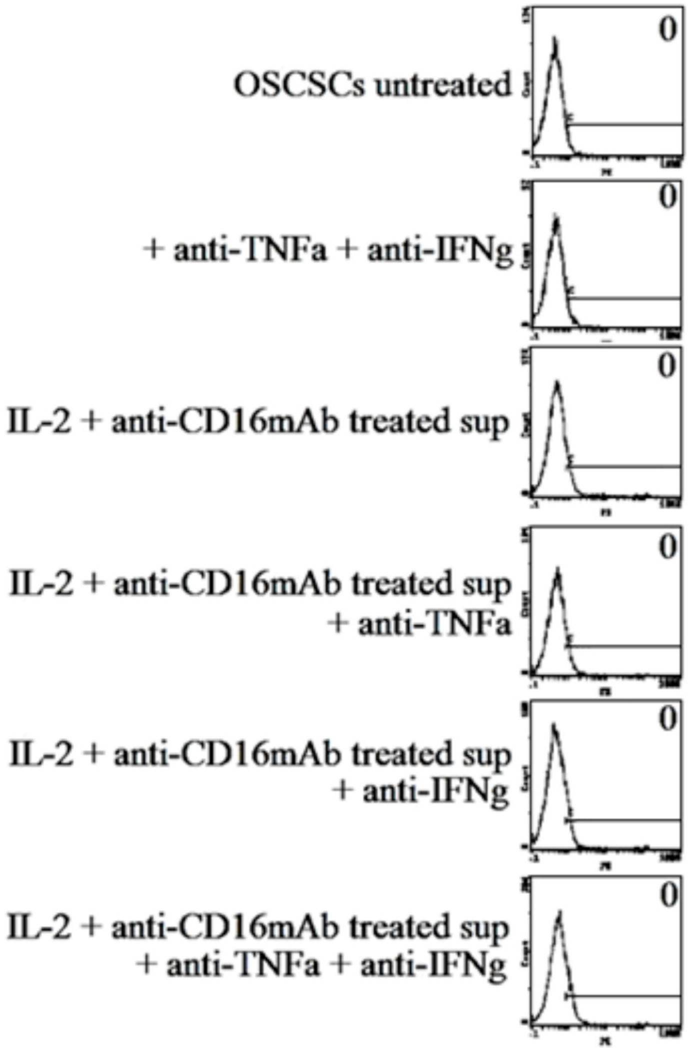

Supplementary Figure 3: Treatment of OSCSCs by supernatants from IL-2 and anti-CD16mAb treated NK cells did not cause cell death. Freshly isolated NK cells were treated with a combination of IL-2 (1000 units/ml) and anti-CD16mAb (3 µg/ml) for 18 hours. After which the supernatants were removed and used for the treatment of OSCSCs in the presence or absence of anti-TNF-α (1:100) and/or anti-IFN-γ (1:100) for a period of 4 days. Untreated OSCSCs and those treated with anti-TNF-α (1:100) and anti-IFN-γ (1:100) in the absence of NK supernatants were used as controls. Afterwards, the viability of OSCSCs was assessed using propidium iodide staining followed by flow cytometric analysis.
